# Pharmacognostic, Phytochemical, and Antioxidant Characterization of *Linaria vulgaris* Mill. Aerial Parts from Central Kazakhstan

**DOI:** 10.3390/plants15213305

**Published:** 2026-10-26

**Authors:** Darkhan Kairatuly Nurkadirov, Zhanar Rakhimovna Shaimerdenova, Gulnur Kurbanalievna Mamytbekova, Yerlan Melsuly Suleimen, Nurgul Bazarkhanovna Askarova, Khorlan Iskozhievna Itzhanova

**Affiliations:** 1 Department of Pharmaceutical Disciplines, Sharmanov National Medical University, Astana Z10K8Y7, Kazakhstan; darhan_2295@mail.ru; 2 School of Pharmacy, NCJSC Karaganda Medical University, Karaganda 100000, Kazakhstan; 3 Department of Chemistry, Chemical Technology and Ecology, JSC “K. Kulazhanov Kazakh University of Business and Information Technologies”, Astana Z05P9M0, Kazakhstan; 4 Department of Normal Physiology, Sharmanov National Medical University, Astana Z10K8Y7, Kazakhstan; nurgulasskar@gmail.com

**Keywords:** *Linaria vulgaris* Mill., pharmacognostic standardization, phytochemical composition, flavonoids, HPLC, amino acids, mineral elements, antioxidant activity

## Abstract

*Linaria vulgaris* Mill. (common toadflax; Plantaginaceae) is a medicinal plant whose pharmacognostic characteristics, chemical composition, and biological activity remain poorly documented. This study aimed to evaluate the aerial parts of *L. vulgaris* growing in Central Kazakhstan (Karaganda Region; collected in July–August 2024) and to provide a scientific basis for the standardization of this medicinal plant material. Resource assessment, pharmacognostic analysis, radiochemical and elemental analyses, HPLC, GC-MS, and in vitro antioxidant assays were performed. The highest raw material yields (232 and 225 kg/ha) were recorded near Molodezhnoye village and in the Karkaraly Mountains, respectively. The main quality parameters were established: loss on drying (6.35 ± 0.07%), total ash (7.37 ± 0.05%), acid-insoluble ash (0.65 ± 0.02%), and extractive content with 70% ethanol (11.34 ± 0.18%). The contents of ^137^Cs and ^90^Sr were below the national regulatory limits, and the analyzed elements showed no substantial contamination; however, a complete safety assessment would also require the determination of mercury, pesticide residues, microbial contamination, and mycotoxins. Phytochemical analysis revealed amino acids and flavonoids, with linarin as the predominant flavonoid (3.91%), indicating its potential as a marker compound for standardization. GC-MS analysis of the essential oil (yield 0.04%) tentatively identified 22 constituents (52.52% of the total peak area), with hexahydrofarnesyl acetone, phytol, palmitic acid, and benzaldehyde as the major ones. The 70% ethanolic extract showed concentration-dependent reducing and DPPH radical-scavenging activities. These findings provide new data on *L. vulgaris* raw material from Central Kazakhstan and support further studies on its quality, standardization, and biological activity in cell-based and in vivo models.

## 1. Introduction

Medicinal plants are valuable sources of biologically active compounds and are widely used to produce herbal medicinal products for the treatment of various chronic diseases. Approximately 20,000 medicinal plant species are known worldwide, accounting for about 4% of all plant species [1]. The flora of Kazakhstan has considerable potential in this respect: of approximately 6400 plant species, more than 700 are used in traditional medicine [2], yet only 67 are included in the Pharmacopeia of Kazakhstan [3,4]. Comprehensive studies of promising local species, covering their resources, quality parameters, and biologically active compounds, are therefore needed to provide a scientific basis for their standardization and possible inclusion in the Pharmacopeia of the Eurasian Economic Union (EAEU).

The genus *Linaria* Mill., which comprises more than 150 taxa, is the largest genus of the tribe *Antirrhineae* and is of considerable pharmacopoeial interest. Owing to the diversity of their secondary metabolites, extracts of *Linaria* species have been reported to exhibit antiproliferative, cytotoxic, antibacterial, anti-inflammatory, antioxidant, and diuretic properties. Nevertheless, the biological potential of individual species remains insufficiently studied [5].

*L. vulgaris* Mill. is a perennial herbaceous plant traditionally assigned to the family Scrophulariaceae and, according to the Angiosperm Phylogeny Group IV classification, now placed in the family Plantaginaceae. The species is native to Europe and temperate Asia, including Kazakhstan and Siberia, and has been introduced into North America and other temperate regions, where it has become a widespread invasive weed [6]. In the flora of Kazakhstan, the genus *Linaria* is represented by 21 species, eight of which are endemic [7].

In traditional medicine, *L. vulgaris* is used to treat digestive and urinary disorders and, externally, purulent wounds and skin rashes [8]. Its anti-inflammatory properties and its use in the treatment of cough and asthmatic conditions have also been reported [9,10]. The species contains various secondary metabolites, including alkaloids, iridoid glycosides, flavonoids, and aurones [9,10,11,12,13]; among them, quinazoline alkaloids (e.g., peganine), the iridoid glycoside antirrhinoside, and flavone glycosides, primarily linarin, are regarded as characteristic of the species and the genus [5,10,13]. The antioxidant activity of a lyophilized *L. vulgaris* infusion has been reported by Czech researchers [14], and flavonoid-rich extracts of the plant have been shown to protect against diet-induced hyperlipidemia and hepatic steatosis in mice [15].

Our previous histochemical studies of *L. vulgaris* growing in Central Kazakhstan revealed, for the first time, flavonoids, phenolic compounds, essential oil components, alkaloids, and sesquiterpene lactones in the leaf and stem tissues, whereas no starch accumulation was detected [16,17].

In addition, the diagnostic morphological characteristics of the stem, leaf, inflorescence, sepals, and corolla of *L. vulgaris* have been established [18].

The chemical composition and biological activity of medicinal plants may vary with the climatic and ecological conditions of their habitat. Despite the wide distribution of *L. vulgaris* in Kazakhstan, no data are available on the raw material resources, pharmacopoeial quality parameters, elemental and amino acid composition, flavonoid profile, essential oil composition, or antioxidant activity of plant material from this region, which hinders the development of regulatory documentation for the species. We hypothesized that natural populations of *L. vulgaris* in Central Kazakhstan provide sufficient raw material resources and that its aerial parts possess a characteristic, linarin-dominated flavonoid profile suitable for standardization. Accordingly, this study aimed to assess the raw material resources of *L. vulgaris* in Central Kazakhstan and to carry out the pharmacopoeial, radiochemical, elemental, phytochemical (amino acids, flavonoids, and essential oil), and in vitro antioxidant characterization of its aerial parts as a basis for the standardization and quality control of this medicinal plant material.

## 2. Results and Discussion

### 2.1. Distribution and Raw Material Resources of Linaria vulgaris Mill. in Central Kazakhstan

*L. vulgaris* is a light-demanding mesoxerophytic species that tolerates water deficit relatively well and is undemanding with respect to soil fertility. It most commonly occurs in dry steppes, on the slopes of low hills, in inter-hill depressions, and on fallow lands.

In the Karaganda Region, the species is widespread and is mainly associated with steppe and meadow habitats, low-hill areas, road margins, scree slopes, and anthropogenically disturbed sites, where it often forms extensive stands.

Route surveys allowed the main areas of distribution of *L. vulgaris* in Central Kazakhstan to be identified. The results of the resource assessment are presented in Table 1.

The highest yields of *L. vulgaris* herb were recorded near Molodezhnoye village (232 kg/ha) and in the Karkaraly Mountains (225 kg/ha), whereas the largest stand area was found in the Nura River floodplain near Botakara village (4.1 ha) (Table 1).

Within the surveyed area, the total exploitable stock of the herb was estimated at 3.58 t and the annual potential harvest at 2.51 t. These preliminary estimates indicate the presence of potentially harvestable populations; however, multi-year monitoring of population recovery and yield variability is required before sustainable harvesting limits can be established.

Thus, *L. vulgaris* is widely distributed in the Karaganda Region and locally forms relatively extensive stands suitable for harvesting. Provided that rational harvesting practices are followed and natural habitats are conserved, the species may serve as a promising source of medicinal plant material for pharmaceutical use.

### 2.2. Pharmacopoeial Evaluation of L. vulgaris Mill. Raw Material

Commodity evaluation is an important stage in the quality assessment of medicinal plant material, as it establishes the identity and quality of the raw material and its compliance with pharmacopoeial requirements.

The commodity evaluation was performed in accordance with pharmacopoeial requirements and included the main quality parameters: loss on drying (LOD), total ash, acid-insoluble ash in 10% hydrochloric acid, and extractive content (Table 2).

Because the efficiency of extraction depends on technological parameters, primarily the choice of solvent and the plant material-to-solvent ratio, the extractive content was determined using ethanol of different concentrations.

The highest extractive yield was obtained with 70% ethanol (11.34 ± 0.18%), whereas 80% and 95% ethanol yielded 8.30 ± 0.14% and 9.19 ± 0.16%, respectively. Preliminary experiments also showed a higher flavonoid yield with 70% ethanol. Therefore, a 70% ethanolic extract obtained by ultrasonic extraction was selected for further phytochemical investigation, and an extraction time of 60 min was chosen on the basis of preliminary experiments.

### 2.3. Safety and Radiation Purity of the Raw Material

#### 2.3.1. Determination of Radionuclides (Cs-137 and Sr-90)

The safe use of medicinal plant materials is limited primarily by the risk of contamination with hazardous substances, including toxic heavy metals, radionuclides, pesticide residues, and microorganisms.

According to current European requirements, the determination of contaminants is an essential quality parameter for ensuring the safety of medicinal plant materials [19]. Particular attention should be paid to radionuclides such as cesium-137 and strontium-90, which have relatively long half-lives (approximately 30 years for ^137^Cs), persist in the environment, and may accumulate in plants. The transfer of radionuclides from plant materials into herbal medicinal products has been reported to range from 24% to 78%. Once in the human body, ^137^Cs accumulates predominantly in muscle tissue, whereas ^90^Sr is deposited in bone tissue; at significant exposure levels, these radionuclides increase the radiation burden and may pose a health risk [20].

Order No. 71 of the Ministry of Healthcare of the Republic of Kazakhstan, dated 2 August 2022, “On Approval of Hygienic Standards for Ensuring Radiation Safety”, sets the permissible levels of radionuclides in medicinal plants at 400 Bq/kg for ^137^Cs and 200 Bq/kg for ^90^Sr. The levels of both radionuclides in the investigated plant material complied with these requirements (Table 3).

The contents of ^137^Cs and ^90^Sr in *L. vulgaris* herb were substantially below these limits, indicating that the investigated sample meets the applicable requirements for radiation safety.

#### 2.3.2. Mineral Composition of L. vulgaris Mill. Medicinal Plant Material

The mineral composition of medicinal plants is of interest both for identifying elements that may contribute to their pharmacological properties and for assessing their safety. Mineral elements are essential for normal physiological processes, whereas toxic elements may accumulate in plant materials as a result of anthropogenic environmental contamination.

The safety of herbal medicinal products has become a particular concern since elevated concentrations of heavy metals were detected in some medicinal plant materials; their presence, especially in traditional medicinal products, may pose a risk to human health and cause adverse effects [21,22]. The elemental composition of the *L. vulgaris* plant material is presented in Table 4.

To evaluate the elemental profile and potential safety of the plant material, the obtained values were compared with regulatory requirements for elemental impurities in medicinal products. According to the EAEU requirements for orally administered products (Decision of the EEC Board No. 138 of 4 October 2022), the indicative permissible concentrations are 0.5 mg/kg for lead (Pb), 0.5 mg/kg for cadmium (Cd), 1.5 mg/kg for arsenic (As), 3.0 mg/kg for mercury (Hg), 20 mg/kg for nickel (Ni), 5 mg/kg for cobalt (Co), and 0.8 mg/kg for thallium (Tl). The values were also compared with the maximum limits for heavy metals in herbal drugs specified in the European Pharmacopeia (1.0 mg/kg for cadmium, 5.0 mg/kg for lead, and 0.1 mg/kg for mercury) [23].

The determined elements were divided into physiologically important mineral elements, including P, Fe, Zn, Mn, and Cu, and potentially toxic elements, namely As, Cd, Pb, Hg, Sb, and Tl.

The contents of As, Cd, antimony (Sb), Tl, uranium (U), and thorium (Th) were below the limits of detection of the analytical method, and Hg was not determined. The Ni content (4.7 mg/kg) was below the reference value of 20 mg/kg for oral administration.

The sample contained macro- and microelements at varying concentrations. The predominant elements were those characteristic of the mineral matrix: phosphorus (2522 mg/kg), iron (453 mg/kg), aluminum (263 mg/kg), strontium (63.4 mg/kg), zinc (45.7 mg/kg), manganese (33.7 mg/kg), and titanium (24 mg/kg).

Phosphorus, the most abundant element (2522 mg/kg), is an essential macronutrient that plays a key role in energy metabolism, nucleic acid biosynthesis, and membrane formation; it is also involved in regulating the uptake, transport, and homeostasis of micronutrients such as iron and zinc [24].

Iron (453 mg/kg) is an essential micronutrient involved in photosynthesis, cellular respiration, and electron transfer, and it is a component of numerous enzymes and iron-containing proteins [25].

The aluminum content was 263 mg/kg. Although aluminum participates in certain physiological processes, its excessive accumulation may exert neurotoxic effects [26]; because it is not an essential element for humans, its accumulation in medicinal plant materials is relevant to safety assessment.

Zinc (45.7 mg/kg) is essential for the functioning of enzymes and regulatory proteins and for plant growth and development, and it may contribute to the nutritional and nutraceutical value of the plant material [27].

Manganese (33.7 mg/kg) is an essential micronutrient involved in enzymatic processes, photosynthesis, and the maintenance of cellular redox homeostasis [28].

Arsenic, cadmium, antimony, thallium, uranium, and thorium were either below the detection limits or present only at trace levels, indicating no significant contamination with these elements.

Other potentially toxic elements were also present at low levels. The tin (Sn) concentration (0.2135 mg/kg) was considerably lower than the reference level of 600 mg/kg established for class 3 elemental impurities in oral medicinal products. The lead content (0.520 mg/kg) was comparable to the indicative limit of 0.5 mg/kg for oral medicinal products and well below the limit of 5.0 mg/kg for herbal drugs in the European Pharmacopeia [23]. Because the EAEU limits apply to finished medicinal products rather than to herbal raw materials, they were used here only for comparison; nevertheless, the lead content should be taken into account in further quality and safety evaluation of this plant material.

Overall, within the sensitivity limits of the analytical method, the results provided no evidence of significant contamination with toxic elements. However, since mercury was not determined, these data do not permit a complete conclusion on the elemental safety of the plant material.

### 2.4. Phytochemical Investigation of Linaria vulgaris Mill.

#### 2.4.1. Quantitative Determination of Amino Acids by GC

Amino acids, together with other phytochemicals and mineral elements, are of considerable interest in cosmetology, as they participate in skin cell signaling pathways that regulate the expression of aging-related genes and help protect cells against various stress factors [29,30].

The acid hydrolysis method allowed most amino acids to be determined (Table 5); tryptophan was not included because its reliable determination requires a separate procedure, such as alkaline hydrolysis.

The aerial parts of *L. vulgaris* showed a diverse amino acid profile. Glutamic acid (2820 mg/100 g) and aspartic acid (1516 mg/100 g) were present at the highest concentrations, indicating a high proportion of acidic amino acids, which play important roles in metabolism.

Among the non-essential amino acids, alanine (862 mg/100 g), proline (480 mg/100 g), serine (362 mg/100 g), and glycine (283 mg/100 g) were present at relatively high concentrations. The relatively high proline content may reflect the response of the plant to environmental stress.

The essential amino acids were represented by leucine (315 mg/100 g), isoleucine (336 mg/100 g), valine (252 mg/100 g), lysine (328 mg/100 g), phenylalanine (316 mg/100 g), threonine (216 mg/100 g), and methionine (65 mg/100 g).

Arginine (415 mg/100 g), histidine (256 mg/100 g), and tyrosine (335 mg/100 g), which are involved in regulatory and metabolic processes, were also present at appreciable concentrations, whereas cystine and hydroxyproline were detected only at trace levels, consistent with their generally low abundance in plant materials.

Overall, these results provide baseline data on the amino acid composition of *L. vulgaris* plant material.

#### 2.4.2. Quantitative Determination of Flavonoids by HPLC

The search for new effective and safe medicinal agents with a broad spectrum of activity and a low risk of adverse effects has drawn particular attention to flavonoids, a class of natural compounds with diverse biological and pharmacological properties.

Flavonoids were selected for quantitative analysis for three reasons: our previous histochemical studies of *L. vulgaris* from Central Kazakhstan identified them as the main phenolic constituents of the leaf and stem tissues [16,17]; linarin, a characteristic flavone glycoside of the genus *Linaria* [5], was considered a candidate marker compound for standardization; and preliminary experiments showed the highest flavonoid yield with 70% ethanol (Section 2.2). Other classes of secondary metabolites, such as phenolic acids and iridoid glycosides, were not quantified in the present study.

The 70% ethanolic extract was therefore analyzed by reversed-phase high-performance liquid chromatography (RP-HPLC), which allowed rutin, linarin, quercetin, and apigenin to be identified and quantified (Figure 1; Table 6).

Linarin (**2**) was the predominant flavonoid of the extract (3.91%), followed by quercetin (**3**, 0.47%), rutin (**1**, 0.37%), and apigenin (**4**, 0.19%) (Figure 2).

The high content of linarin is of particular pharmacological interest, as this compound has been reported to exert pronounced anti-inflammatory and antioxidant effects. Linarin reduces the production of pro-inflammatory mediators, including NO, PGE2, IL-6, and TNF-α, and suppresses the expression of COX-2 and iNOS [31,32]; moreover, its protective effect against ischemia–reperfusion injury has been associated with activation of the Nrf2 and PI3K/Akt signaling pathways and enhancement of antioxidant defense [33].

The predominance of linarin may therefore contribute to the biological activity of the extract. Nevertheless, the overall effects of the extract are likely to result from the combined action of several constituents, including phenolic acids, iridoid glycosides, and other compounds that were not quantified in the present study.

The predominance of linarin is consistent with literature data, as this flavone glycoside has been isolated from several *Linaria* species, including *L. vulgaris*, *L. japonica*, and *L. kurdica* subsp. *eriocalyx* [34]. In *Chrysanthemum indicum*, one of the best-studied sources of linarin, its content reached 1.47 g/100 g in the leaves and 0.65 g/100 g in the stems and flowers [34]. Although direct comparison is limited by differences in plant material and in the basis of calculation, these data confirm that linarin is a characteristic constituent of *L. vulgaris* and support its potential use as a marker compound for standardization.

The flavonoid profile may also be partly related to the ecological conditions of the collection site. *L. vulgaris* is a light-demanding mesoxerophyte, and the sharply continental climate of Central Kazakhstan, with high summer insolation and water deficit, together with the higher-than-normal summer temperatures in the collection year (Section 3.1), may favor the accumulation of flavonoids, which act as UV-protective and antioxidant metabolites in plant tissues [35]. Similarly, the relatively high proline content (Table 5) may reflect the response of the plant to drought stress, as proline is a well-known osmoprotectant [36]. These assumptions, however, require verification in comparative studies of populations from different habitats.

#### 2.4.3. Qualitative and Quantitative Composition of the Essential Oil of *L. vulgaris* Mill.

The analysis of essential oils, including the identification of their constituents and the isolation of major compounds for standardization purposes, remains a promising area for the domestic pharmaceutical industry.

The essential oil obtained by hydrodistillation was a dark-green liquid with a yield of 0.04% (*w*/*w*) on an air-dried basis.

Its chemical composition was investigated by gas chromatography–mass spectrometry (GC-MS) at the Research Center of NJSC “Karaganda Medical University” (Karaganda, Kazakhstan); the identified constituents are presented in Table 7, and the chromatogram is shown in Figure 3.

RI, linear retention index on the HP-5MS column. All constituents were tentatively identified by comparison of their mass spectra and retention indices with library data [37]; authentic standards were not analyzed.

In total, 22 compounds were tentatively identified, accounting for 52.52% of the total chromatographic peak area, whereas 47.48% remained unidentified. The major identified constituents were hexahydrofarnesyl acetone (**6**) 11.71%, phytol (**8**) 11.53%, palmitic acid (**7**) 6.82%, and benzaldehyde (**5**) 6.75% (Figure 4).

The oil was chemically diverse and contained aliphatic and aromatic aldehydes, carboxylic acids, norisoprenoids, sesquiterpenes, sesquiterpenoids, alcohols, and phenolic compounds. Aliphatic alcohols, including (*Z*)-hex-3-en-1-ol, *n*-hexanol, oct-1-en-3-ol, and octan-3-ol, together with decanoic and palmitic acids, further contributed to its characteristic chemical profile.

These data expand the available information on the essential oil of *L. vulgaris* and may be useful for its identification, for comparison with related plant materials, and for developing quality and authenticity criteria.

Data on the essential oils of the genus *Linaria* are scarce. In the oil of *L. ventricosa*, the main constituent was 4-vinylguaiacol (17.4%) [38], whereas in the *L. vulgaris* oil it accounted for only 1.08%. In contrast, hexahydrofarnesyl acetone, phytol, and palmitic acid, which predominated in our sample, were among the principal volatile compounds of 18 *Veronica* species (Plantaginaceae) [39] and of the autumn oil of *Scrophularia frigida* (6.65%, 12.99%, and 30.49%, respectively), whereas the summer oil of the latter was dominated by linalool [40]. Since volatile profiles depend strongly on environmental factors and developmental stage [39,40], these factors may partly explain the differences from other *Linaria* species. Benzaldehyde (6.75%), one of the most widespread floral scent compounds [41], probably originated partly from the flowers, as the plants were collected at full flowering. Phytol has been reported to possess antioxidant, antimicrobial, and anti-inflammatory activities [42], although its contribution is likely to be limited given the low oil yield (0.04%).

Almost half of the total peak area (47.48%) remained unassigned because the mass spectra of the corresponding peaks showed no reliable match with library and retention-index data. These results therefore represent a preliminary profile rather than a complete characterization of the oil, which, to our knowledge, has not previously been reported for *L. vulgaris*. Moreover, because the essential oil and the 70% ethanolic extract are different fractions of the plant material, the antioxidant activity of the extract (Section 2.5) is not attributed to the oil constituents.

### 2.5. Antioxidant Activity of L. vulgaris Mill. Extract

Flavonoids exhibit a broad spectrum of biological activities, including antioxidant, anti-inflammatory, and cardioprotective effects, and are considered promising agents for the prevention of various diseases [43,44]. The flavonoid profile and antioxidant properties of medicinal plants are therefore of considerable scientific interest.

The antioxidant capacity of the 70% ethanolic extract of *L. vulgaris* was evaluated using two complementary chemical assays. The reducing-power assay was used to assess the ability of the extract to reduce Fe^3+^ to Fe^2+^, whereas the DPPH assay was employed to evaluate its free-radical-scavenging capacity. As these assays reflect different aspects of antioxidant activity, their combined use provides a broader preliminary assessment of the in vitro antioxidant capacity of the extract.

#### 2.5.1. Reducing-Power Activity

The 70% ethanolic extract of *L. vulgaris* exhibited a concentration-dependent ferric-reducing capacity. The absorbance at 700 nm increased from 0.7066 ± 0.012 at 0.25 mg/mL to 1.2124 ± 0.02 at 1.0 mg/mL (Table 8; Figure 5), indicating an increased ability of the extract to participate in electron-transfer reactions as its concentration increased. Nevertheless, at all tested concentrations, the reducing capacity of the extract remained lower than that of ascorbic acid, for which absorbance values ranged from 1.925 ± 0.01 to 2.316 ± 0.01.

Ferric-reducing activity has also been reported for several other species of the genus *Linaria*. Mouffouk et al. observed antioxidant and ferric-reducing activity in the methanolic extract of *L. scariosa* [45]. Similarly, Gul et al. demonstrated reducing activity in different extracts of *L. corifolia*, with the ethanolic extract of the aerial parts showing comparatively high antioxidant activity and relatively high phenolic and flavonoid contents [46]. Cheriet et al. reported reducing-power activity for hydroalcoholic and ultrasound-assisted extracts of *L. reflexa*, both of which were characterized by a glycosylated flavonoid-rich profile [47]. Furthermore, *L. polygalifolia* subsp. *polygalifolia* showed an FRAP value of 0.103 mmol AAE/g dry extract, and a significant correlation between total phenolic content and FRAP (r = 0.685) was observed across the plant extracts examined in that study [48].

Collectively, these findings are consistent with the concentration-dependent ferric-reducing response observed for the 70% ethanolic extract of *L. vulgaris* in the present study and demonstrate considerable variation in reducing capacity among *Linaria* species. Such variation may reflect differences in species-specific phytochemical composition as well as extraction conditions and solvent polarity.

The reducing capacity observed in the present study may be associated, at least in part, with the phenolic constituents of the 70% ethanolic extract. HPLC analysis identified linarin as the predominant flavonoid (3.91%), together with smaller amounts of quercetin (0.47%), rutin (0.37%), and apigenin (0.19%). The reducing properties of flavonoids are strongly influenced by their molecular structure, including the number and position of hydroxyl groups, conjugation within the flavonoid skeleton, and the degree of glycosylation and methoxylation. Therefore, the quantitative predominance of linarin should not be interpreted as evidence that it is the principal contributor to the observed reducing response. Quercetin, rutin, apigenin, other phenolic constituents not quantified in the present study, and possible interactions among extract components may collectively contribute to the measured activity. Since the individual compounds and their defined mixtures were not evaluated separately, their relative contributions cannot be established from the present data. Accordingly, the reducing-power response should be interpreted as the overall ferric-reducing capacity of the extract rather than as an effect attributable to a specific constituent.

#### 2.5.2. DPPH Radical-Scavenging Activity

The 70% ethanolic extract of *L. vulgaris* exhibited concentration-dependent DPPH radical-scavenging activity. The inhibition increased from 27.05% at 0.25 mg/mL to 62.50% at 0.50 mg/mL, 70.68% at 0.75 mg/mL, and 76.65% at 1.00 mg/mL. The greatest increase was observed between 0.25 and 0.50 mg/mL, followed by a more gradual increase at higher concentrations. At 1.00 mg/mL, the extract showed 76.65% inhibition, approaching the activity of BHA under the same experimental conditions (Table 9; Figure 6).

Similar DPPH radical-scavenging activity has been reported for other *Linaria* extracts. Vrchovská et al. demonstrated concentration-dependent DPPH scavenging in an infusion of *L. vulgaris* [14]. Antioxidant activity was also reported for extracts of *L. corifolia* by Gul et al. [46] and for hydroalcoholic and ultrasound-assisted extracts of *L. reflexa* by Cheriet et al. [47]. In contrast, the aqueous extract of *L. simplex* showed relatively weak DPPH-scavenging activity [49], while *L. scariosa* also exhibited measurable radical-scavenging activity [45]. These findings indicate that DPPH-scavenging capacity is common among *Linaria* species, although its magnitude varies among species and extracts.

Overall, the present results demonstrate the concentration-dependent DPPH radical-scavenging activity of the 70% ethanolic extract of *L. vulgaris*. However, these findings are based on an in vitro chemical assay and require further confirmation in biological models.

## 3. Materials and Methods

### 3.1. Plant Collection

The aerial parts of *L. vulgaris* were collected during the full flowering period (July–August 2024) from natural habitats in the Sogety tract, Bukhar-Zhyrau District, Karaganda Region, Republic of Kazakhstan (50.064529° N, 73.446468° E). Collection and primary processing were carried out in accordance with the principles of Good Agricultural and Collection Practices (GACPs).

The plant material was identified by Prof. Margarita Yu. Ishmuratova, Candidate of Biological Sciences (Karaganda National Research University named after E.A. Buketov), and a voucher specimen (No. QAR 00374) has been deposited in the Herbarium of the same university (Karaganda, Kazakhstan).

The climate of the collection area is sharply continental. According to the 1991–2020 climatic norms of the nearest meteorological station, Karaganda (49.80° N, 73.15° E, 553 m a.s.l.), located approximately 36 km south–west of the collection site, the mean annual air temperature is 3.2 °C, the mean January and July temperatures are −14.2 °C and 20.2 °C, respectively, and the mean annual precipitation is 380 mm, with a maximum in June–July [50]. The collection season was warmer than usual: the mean air temperatures in July and August 2024 (23.3 °C and 20.9 °C) were 3.1 °C and 2.8 °C above the norms [51]. Detailed soil analysis was not performed in the present study.

After collection, the plant material was sorted to remove mechanical and biological impurities, dried naturally in a well-ventilated room protected from direct sunlight (25 ± 2 °C; relative humidity 60 ± 5%), milled to a particle size of 2–3 mm, and stored in tightly closed containers at room temperature until analysis.

### 3.2. Assessment of Raw Material Resources

The resources of *L. vulgaris* aerial parts were assessed by the sample-plot method described by Elzinga et al. [52]. At each study site, 10–15 sample plots of 1 m^2^ were established, and the aerial parts were cut at a height of 5 cm above the soil surface, dried to an air-dry state, and weighed. The stand area was determined by measuring its length and width. The mean yield per sample plot was converted to kg/ha, the exploitable stock was calculated as the product of the mean yield and the total stand area, and the potential annual harvest was estimated as 70% of the exploitable stock.

### 3.3. Pharmacopoeial Evaluation of L. vulgaris Mill. Raw Material

The pharmacopoeial evaluation was performed according to the State Pharmacopeia of the Republic of Kazakhstan (SP RK, Vol. 1) and included the determination of loss on drying, total ash, acid-insoluble ash in 10% hydrochloric acid, and extractive content; each parameter was determined in three independent measurements [53].

### 3.4. Determination of Radiation Purity and Safety Indicators

#### 3.4.1. Determination of Radionuclides

The concentrations of ^137^Cs and ^90^Sr were determined by radiochemical methods followed by β-spectrometric analysis at the EcoExpert Testing Center (Karaganda, Kazakhstan) according to MIK KZ.07.00316-2015 and MIK KZ.07.00315-2015, respectively, without preliminary ashing of the samples. The obtained values were compared with the permissible levels established by the State Pharmacopeia of the Republic of Kazakhstan [53].

#### 3.4.2. Determination of the Elemental Composition

The elemental composition was determined by inductively coupled plasma optical emission spectrometry (ICP-OES) on a Leeman Lab Profile Plus spectrometer at the Chemical Analytical Laboratory of Azimut Geology LLP (Karaganda, Kazakhstan) according to the methodology MVI KZ.01.037178-2016, in compliance with the State Pharmacopeia of the Republic of Kazakhstan, Vol. 1, 2nd edition, General Pharmacopoeial Monograph 2.2.23 [53].

### 3.5. Extract Preparation

Air-dried aerial parts (50.0 g; particle size 2–3 mm) were extracted with 500 mL of 70% ethanol (plant material-to-solvent ratio 1:10, *w*/*v*) in a VGT-1200 ultrasonic bath (GT SONIC, Shenzhen, China) at 40 kHz and 180 W at 25 ± 2 °C for 60 min; the extraction was repeated three times [54].

The combined extracts were filtered, concentrated under reduced pressure on a rotary evaporator until complete removal of the solvent, and dried to constant weight. The extraction yield was expressed as a percentage of the mass of the air-dried plant material.

### 3.6. Phytochemical Analysis

#### 3.6.1. Determination of Amino Acid Composition

The amino acid composition of the aerial parts of *L. vulgaris* Mill., collected during the flowering stage and air-dried in the shade, was determined by gas–liquid chromatography (GLC) as N-acetyl n-propyl ester derivatives, according to the method described by Adams [55]. For the analysis, 1.0 g (accurately weighed) of powdered plant material was hydrolyzed with 5 mL of 6 N HCl at 105 °C for 24 h in sealed ampoules under an argon atmosphere. The hydrolysate was evaporated to dryness under vacuum at 40–50 °C, after which the dry residue was dissolved in 5 mL of sulfosalicylic acid solution. Following centrifugation at 1500 rpm for 5 min, the supernatant was applied to a Dowex 50, H-8, 200–400 mesh ion-exchange resin column. The column was washed with deionized water and 0.5 N acetic acid solution until a neutral pH was reached. Amino acids were eluted with 3 mL of 6 N NH_4_OH, after which the eluate was evaporated to dryness at 40–50 °C.

For derivatization, one drop of 1.5% SnCl_2_ solution, one drop of 2,2-dimethoxypropane, and 1–2 mL of HCl-saturated propanol were added to the dry residue. The mixture was heated at 110 °C for 20 min and evaporated. Subsequently, 1 mL of acetylating reagent (acetic anhydride:triethylamine:acetone, 1:2:5) was added, and the mixture was heated at 60 °C for 1.5–2 min. After evaporation, the residue was extracted with 2 mL of ethyl acetate in the presence of 1 mL of saturated NaCl solution. The upper ethyl acetate layer was used for GLC analysis.

Chromatographic analysis was performed using a Carlo Erba 4200 gas–liquid chromatograph (Italy–USA) equipped with a flame ionization detector. The detector and injector temperatures were 300 °C and 250 °C, respectively. The column temperature was programmed from 110 to 185 °C at a rate of 18.6 °C/min and subsequently to 250 °C at a rate of 32 °C/min; the temperature was maintained at 250 °C until complete elution of the analyzed components. Separation was performed on a stainless-steel column (400 × 3 mm) packed with a stationary phase containing 0.31% Carbowax 20M, 0.28% Silar 5CP, and 0.06% Lexan on Chromosorb W-AW, 120-140 mesh. Quantification was performed using the external standard method with standards supplied by Altex.

#### 3.6.2. Quantitative Determination of Flavonoids by HPLC

The flavonoids quercetin, rutin, linarin, and apigenin in the *L. vulgaris* Mill. extract were quantitatively determined by reversed-phase high-performance liquid chromatography (RP-HPLC) using a WOOKING K2025 HPLC system (Wooking Scientific Instruments (Shanghai) Co., Ltd., Shanghai, China) equipped with a UV detector. Separation was performed under isocratic conditions using a Kromasil 100-5-C_18_ column (4.6 × 250 mm, 5 µm) at a temperature of 40 °C. The mobile phase consisted of acetonitrile and 1% acetic acid solution at a ratio of 50:50 (*v*/*v*), with a flow rate of 1.0 mL/min. The injection volume was 20 µL, and detection was performed at a wavelength of 280 nm.

Prior to analysis, the thick *L. vulgaris* Mill. extract was dissolved in the mobile phase and filtered through a membrane filter with a pore size of 0.22 µm. The compounds were identified by comparing the retention times of the sample peaks with those of quercetin, rutin, linarin, and apigenin reference standards. Quantification was performed using the external standard method based on calibration curves constructed from standard solutions of the corresponding compounds.

Each sample was analyzed in triplicate (*n* = 3), and consistent results were obtained in repeated analyses. A full validation of the HPLC method in accordance with ICH Q2(R2) (linearity, limits of detection and quantification, precision, accuracy, and robustness) was not performed in the present study; therefore, the obtained flavonoid contents should be regarded as preliminary. The validation of the method for linarin as a candidate marker compound is planned as the next stage of the standardization of *L. vulgaris* medicinal plant material.

#### 3.6.3. Essential Oil Isolation

Essential oil from 500 g of the aerial parts of *L. vulgaris* Mill. was obtained by hydrodistillation using a Clevenger-type apparatus in accordance with the requirements of the European Pharmacopeia [23]. Distillation was carried out for 3 h until no further increase in the volume of the collected essential oil was observed [56]. A separate dark-green oil phase with a mass of 0.20 g was obtained, corresponding to an essential oil yield of 0.04% (*w*/*w*) based on the mass of the air-dried plant material.

The essential oil was dried over anhydrous sodium sulfate and stored in tightly closed dark glass vials at −20 °C until GC-MS analysis (Section 3.6.4).

#### 3.6.4. GC-MS Analysis of Essential Oil

The chemical composition of the essential oil of *L. vulgaris* Mill. was investigated by gas chromatography–mass spectrometry (GC-MS) using an Agilent 7890B gas chromatograph coupled to an Agilent 5977B mass-selective detector (Agilent Technologies, Santa Clara, CA, USA). Separation was performed on an HP-5MS capillary column (5% phenyl methyl siloxane; 30 m × 0.25 mm) using helium as the carrier gas at a flow rate of 1.0 mL/min. The injector temperature was 280 °C. The oven temperature was initially maintained at 50 °C for 2 min, then increased to 280 °C at a rate of 4 °C/min and held at 280 °C for 20 min. The sample was injected in splitless mode, with an injection volume of 0.2 µL. Mass spectra were recorded using electron ionization at 70 eV over an *m*/*z* range of 10–350.

The components were identified by comparing their mass spectra and linear retention indices with data from the database of the N.N. Vorozhtsov Novosibirsk Institute of Organic Chemistry, Siberian Branch of the Russian Academy of Sciences (NIOCH SB RAS) [37], as well as with published literature data. Linear retention indices were calculated relative to n-alkanes (C_7_–C_40_). The relative composition was determined by the internal normalization method based on the peak areas of the chromatographic components using Agilent MSD ChemStation software (version E.02.02, Agilent Technologies, Santa Clara, CA, USA), without applying correction factors. The relative content of each identified component was calculated as a percentage of the total integrated chromatographic peak area. Unidentified peaks were included in the total peak-area normalization but were not included in the component identification table. Because authentic standards were not analyzed, all compounds are reported as tentatively identified.

### 3.7. Determination of Antioxidant Activity

#### 3.7.1. Reducing-Power Assay

The reducing capacity of the samples was evaluated using the potassium ferricyanide reduction method, based on the ability of antioxidants to reduce Fe^3+^ to Fe^2+^ [57,58].

For the analysis, 1.0 mL of sample solutions at concentrations of 0.25, 0.50, 0.75, and 1.00 mg/mL was mixed with 2.5 mL of phosphate buffer (0.2 M, pH 6.6) and 2.5 mL of 1% potassium ferricyanide (K_3_[Fe(CN)_6_]) solution. The reaction mixture was incubated at 50 °C for 25 min. After incubation, the reaction was terminated by adding 2.5 mL of 10% trichloroacetic acid, followed by centrifugation at 3000 rpm for 3 min. Subsequently, 2.5 mL of the supernatant was mixed with 2.5 mL of distilled water and 0.5 mL of 0.1% ferric chloride (FeCl_3_) solution. The mixture was allowed to stand at room temperature for 10 min, after which the absorbance was measured at 700 nm using an Agilent Cary 60 UV-Vis spectrophotometer (Agilent Technologies, Santa Clara, CA, USA). Ascorbic acid was used as the reference antioxidant.

#### 3.7.2. DPPH Assay

The antiradical activity of the samples was determined based on their ability to scavenge the stable free radical 2,2-diphenyl-1-picrylhydrazyl (DPPH) [56,58,59]. The samples were dissolved in ethanol to obtain solutions at concentrations of 0.25, 0.50, 0.75, and 1.00 mg/mL. Then, 0.1 mL of the sample solution was added to 3.0 mL of freshly prepared 0.06 mM DPPH solution in ethanol. The mixtures were thoroughly mixed and incubated in the dark at room temperature for 30 min.

After incubation, the absorbance was measured at 517 nm against a control solution (DPPH solution without the sample) using an Agilent Cary 60 UV–Vis spectrophotometer (Agilent Technologies, Santa Clara, CA, USA).

The percentage of DPPH radical inhibition was calculated using the following equation:

ARA(%) = [(A_0_ − A_t_)/A_0_] × 100 (1)

where A_0_ is the absorbance of the control solution and A_t_ is the absorbance of the sample. Butylated hydroxyanisole (BHA) was used as the positive control.

### 3.8. Statistical Analysis

All experiments were performed in triplicate, and the results are presented as the mean ± SD. Statistical analysis was performed using GraphPad Prism version 10 (GraphPad Software, San Diego, CA, USA). Differences among concentrations and between the extract and reference antioxidant were evaluated by one-way ANOVA followed by the Bonferroni post hoc test. Differences were considered statistically significant at *p* < 0.05.

## 4. Conclusions

This study provides the first comprehensive characterization of *L. vulgaris* plant material from Central Kazakhstan as a scientific basis for its standardization. The main areas of occurrence and the raw material resources of the species were identified, with an estimated annual potential harvest of 2.51 t. The contents of ^137^Cs and ^90^Sr were below the national regulatory limits, and the analyzed potentially toxic elements were below the limits of detection or the reference values; however, a complete safety assessment would also require the determination of mercury, pesticide residues, microbial contamination, and mycotoxins.

The aerial parts contained amino acids and flavonoids, with linarin as the predominant flavonoid (3.91%), supporting its potential use as a chemical marker for the identification and standardization of *L. vulgaris* plant material. A preliminary profile of the essential oil was obtained for the first time, with hexahydrofarnesyl acetone and phytol as the major tentatively identified constituents, although 47.48% of the total peak area remained unidentified.

The 70% ethanolic extract showed concentration-dependent reducing and radical-scavenging capacity, which requires confirmation in biological models. Taken together, the resource, pharmacopoeial, chemical, and in vitro antioxidant data provide a scientific basis for developing regulatory documentation and quality and standardization parameters for this plant material.

The main limitations of the study are the analysis of plant material from a single site and season, the incomplete safety assessment, the tentative identification of the essential-oil constituents with a substantial unidentified fraction, and the use of chemical in vitro antioxidant assays only. Future studies should focus on the validation of an HPLC (and LC-MS/MS) method for linarin as a marker compound, a complete safety assessment, multi-year monitoring of natural populations, and pharmacological studies in cell-based and in vivo models.

## Figures and Tables

**Figure 1:**
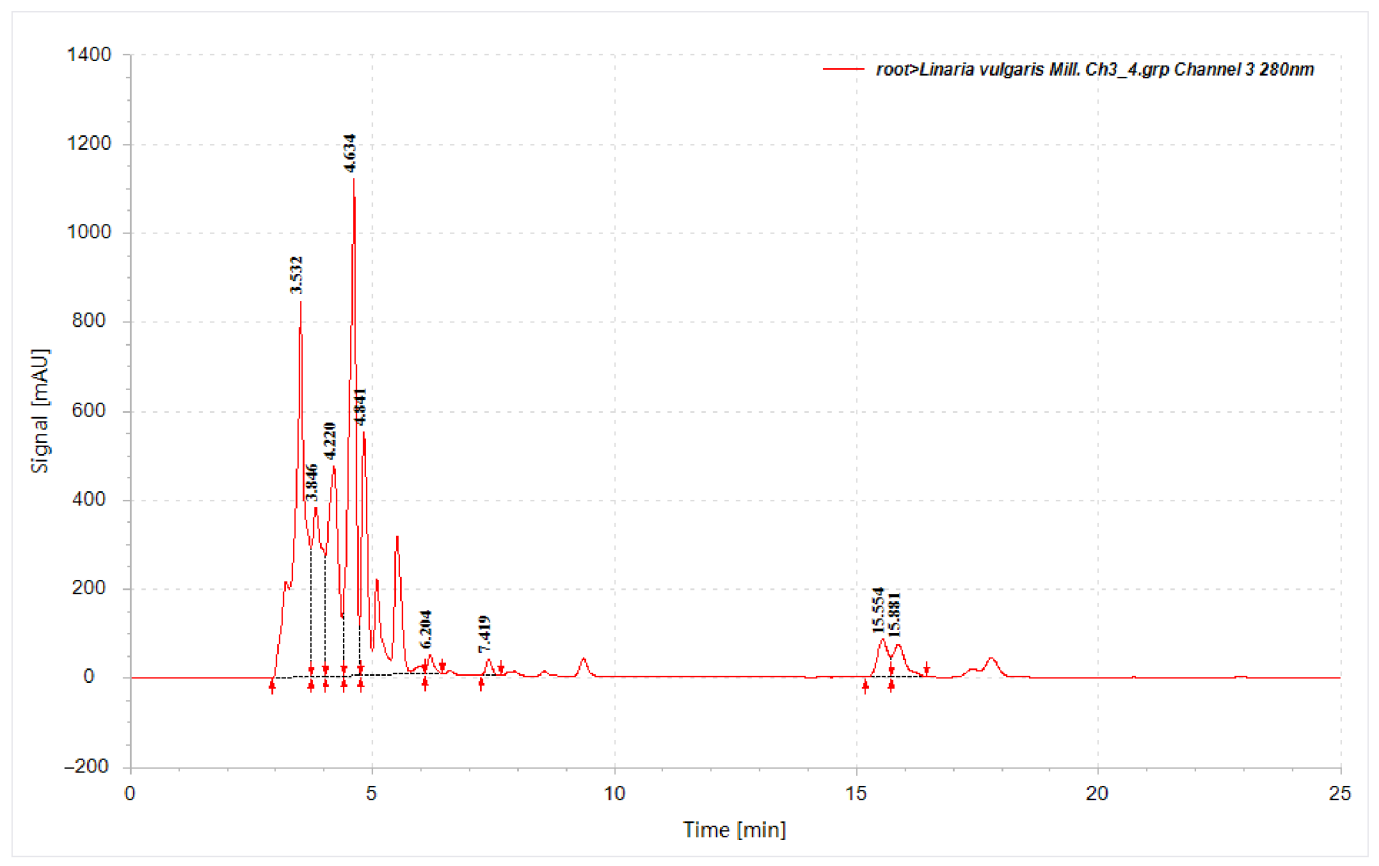
HPLC chromatogram of the total extractive fraction of *L. vulgaris* Mill.

**Figure 2:**
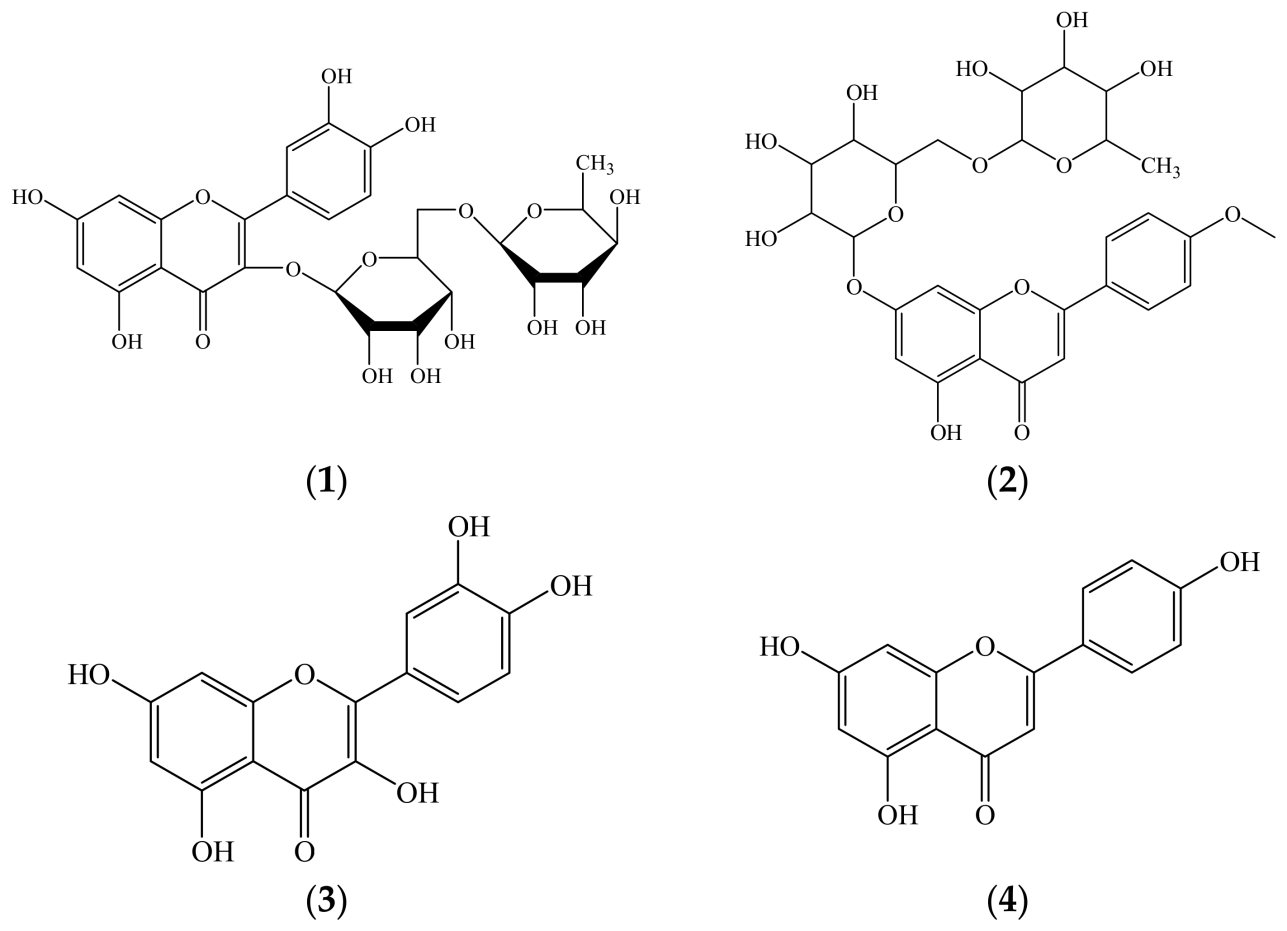
Chemical structures of the flavonoids identified in the *L. vulgaris* Mill. extract: rutin (**1**), linarin (**2**), quercetin (**3**), and apigenin (**4**).

**Figure 3:**
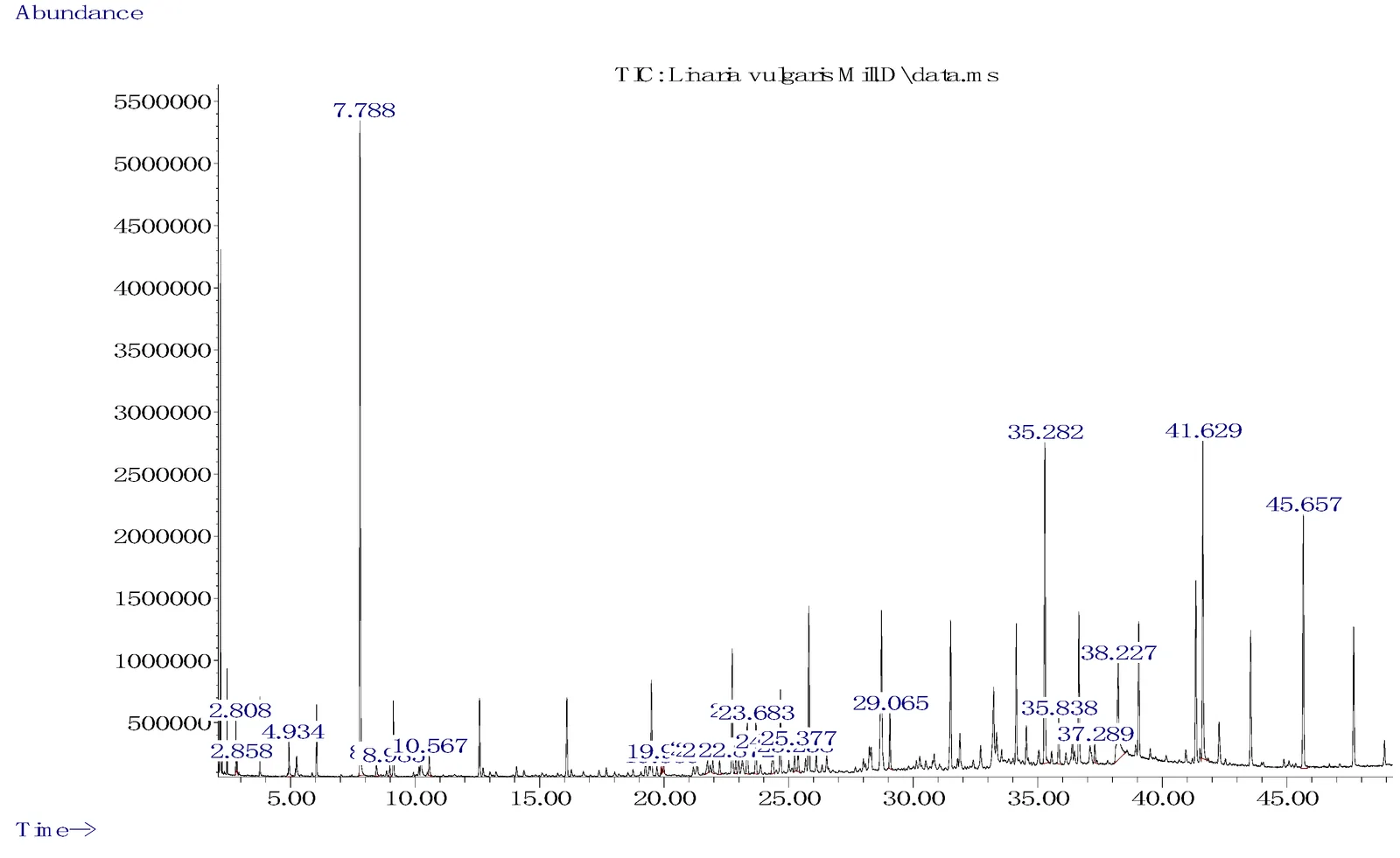
GC-MS chromatogram of the essential oil composition of *L. vulgaris* Mill. (0–50 min region; no peaks were detected after 50 min).

**Figure 4:**
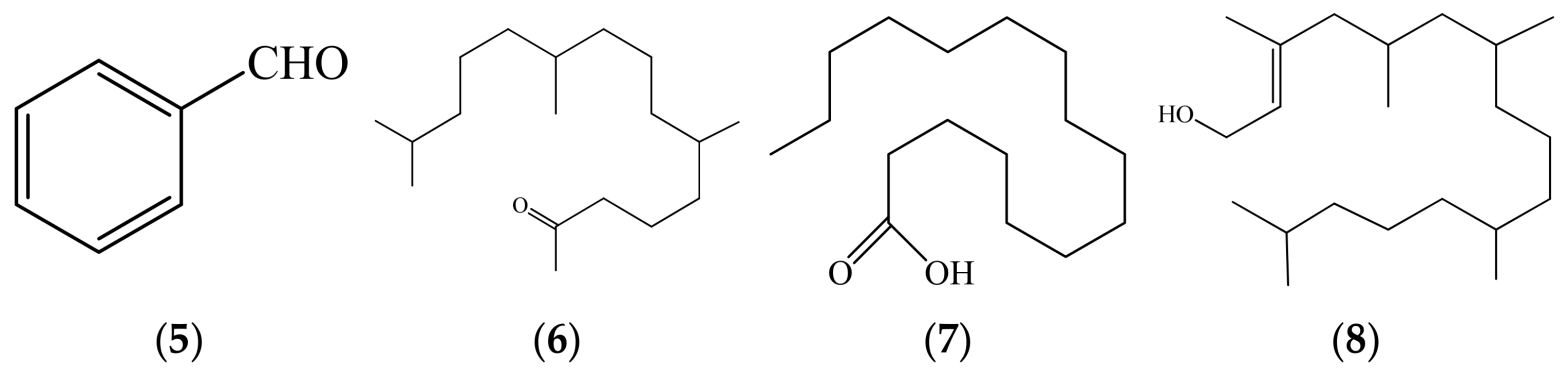
Chemical structures of the major constituents of the essential oil of *L. vulgaris* Mill.: benzaldehyde (**5**), hexahydrofarnesyl acetone (**6**), palmitic acid (**7**), and phytol (**8**).

**Figure 5:**
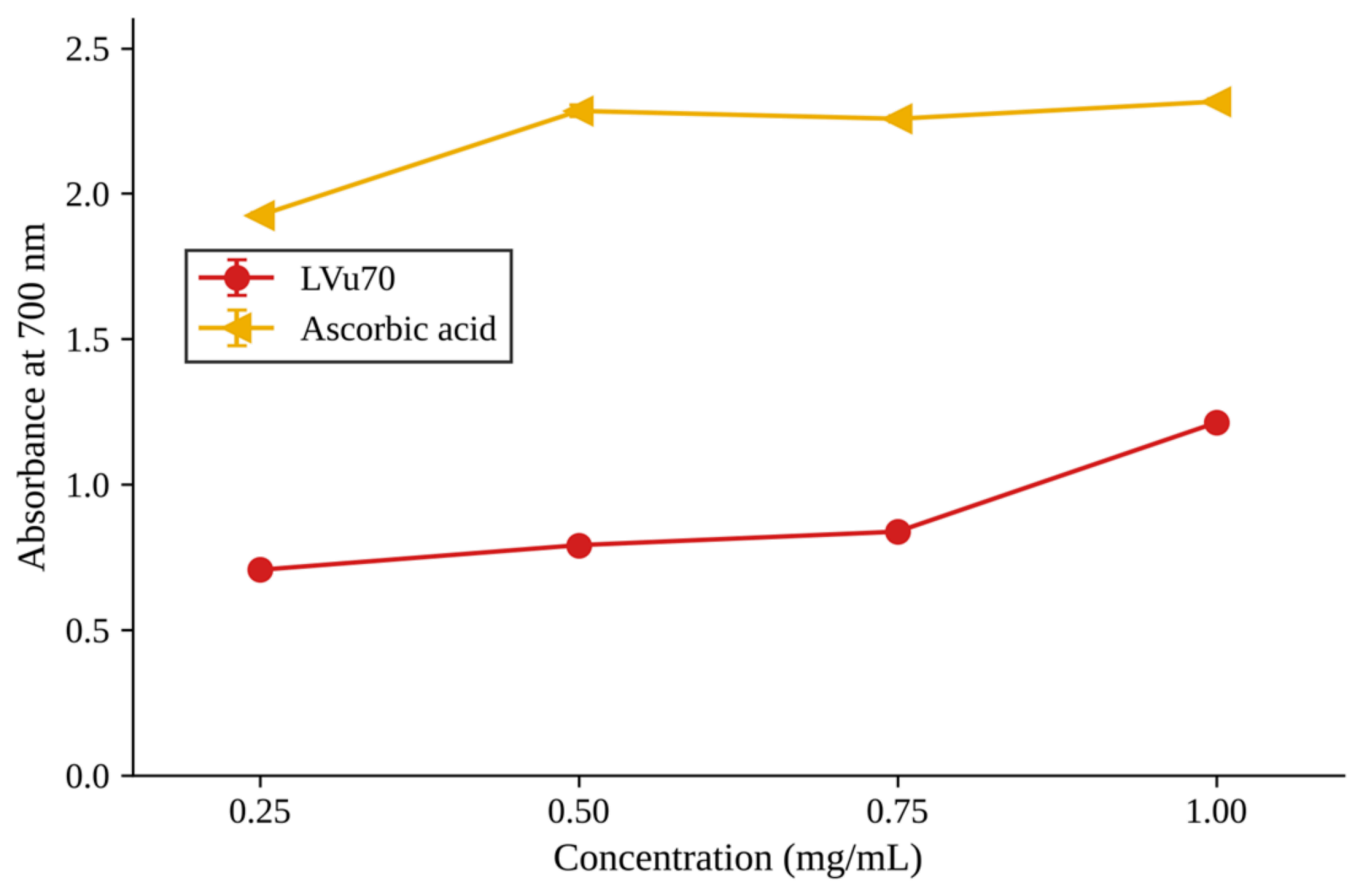
Dependence of absorbance on the concentration of the investigated substances.

**Figure 6:**
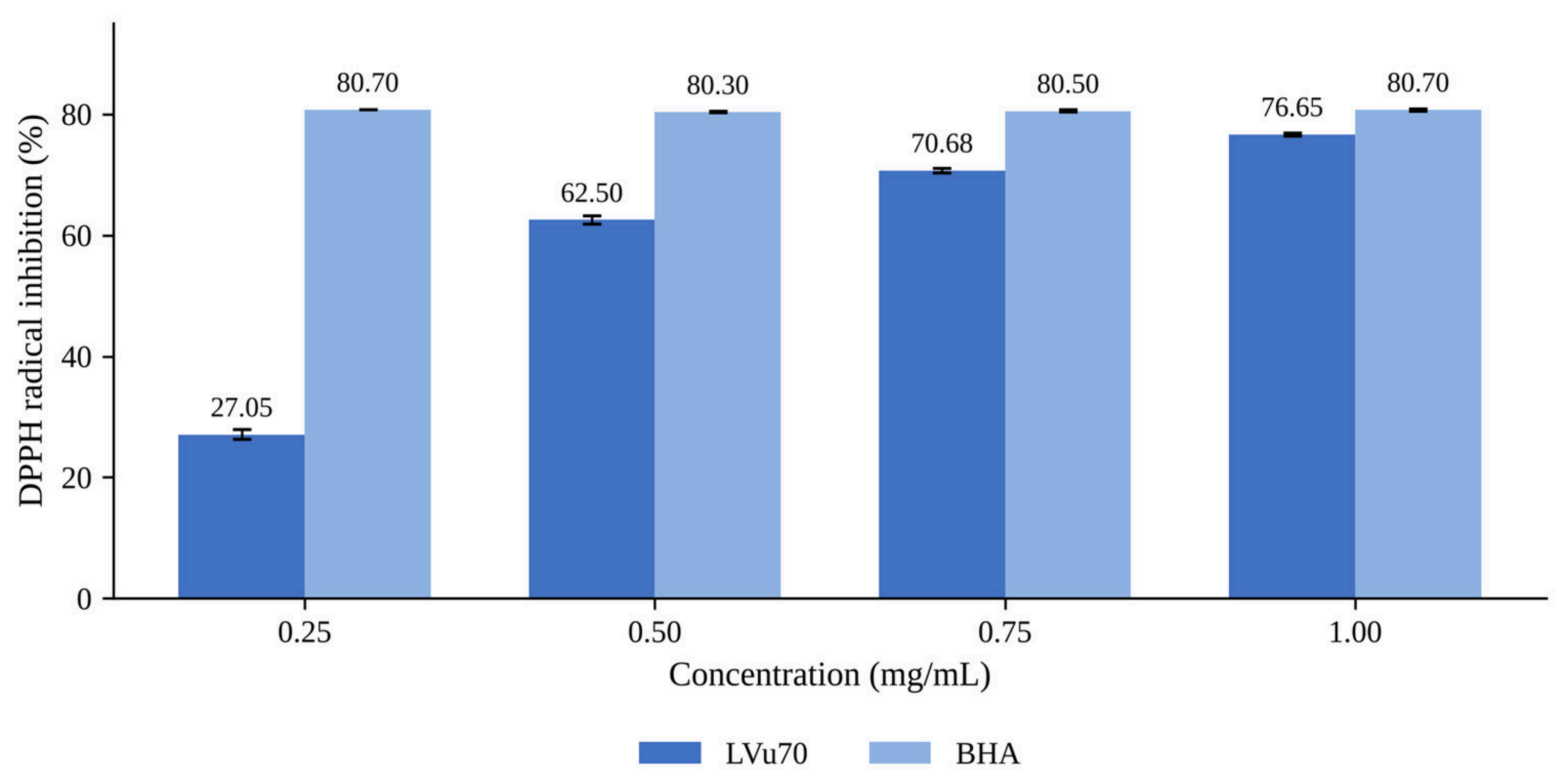
Dependence of the antiradical activity of *L. vulgaris* Mill. extract on its concentration.

**Table 1:** Stand area, yield, exploitable stock, and annual potential harvest of *L. vulgaris* Mill. herb at six localities in the Karaganda Region (Central Kazakhstan).

Location	Stand Area (ha)	Yield (kg/ha)	Exploitable Stock (t)	Annual Potential Harvest (t)
Karkaraly Mountains	2.7	225	0.61	0.42
Nura River floodplain near Botakara village	4.1	210	0.86	0.60
Vicinity of Kulaygyr village	3.0	180	0.54	0.38
Vicinity of Molodezhnoye village	2.9	232	0.67	0.47
Steppe area beyond Karabas village	3.2	165	0.53	0.37
Vicinity of Kerney village	1.8	215	0.38	0.27
Total	17.7		3.58	2.51

**Table 2:** Pharmacopoeial quality parameters of *L. vulgaris* Mill. herb and extractive content obtained with ethanol of different concentrations (mean ± SD, *n* = 3).

Parameters	Content (% *w*/*w*, Mean ± SD)
Loss on drying (LOD)	6.35 ± 0.07
Total ash	7.37 ± 0.05
Acid-insoluble ash in 10% hydrochloric acid	0.65 ± 0.02
Extractives-95% ethanol	9.19 ± 0.16
Extractives-80% ethanol	8.30 ± 0.14
Extractives-70% ethanol	11.34 ± 0.18
Extractives-50% ethanol	8.71 ± 0.12

**Table 3:** Contents of ^137^Cs and ^90^Sr in *L. vulgaris* Mill. herb compared with the national regulatory limits.

Parameter	Regulatory Limit (Bq/kg)	Obtained Value (Bq/kg)
^137^Cs	400	<1.8
^90^Sr	200	<12

**Table 4:** Elemental composition of *L. vulgaris* Mill. herb collected in Central Kazakhstan, determined by ICP-OES.

Elements Determined	Content (mg/kg)	Elements Determined	Content (mg/kg)
Ag	<0.1	Mo	<0.1
Al	263	Nb	<0.1
As	<0.1	Ni	4.7
B	<1	P	2522
Ba	10	Pb	0.520
Be	<0.05	Sb	<0.1
Bi	<0.1	Sc	<0.1
Cd	<0.05	Se	<0.1
Ce	0.526	Sn	0.2135
Co	<0.1	Sr	63.4
Cr	8.1	Tc	<0.1
Cu	8.1	Th	<0.05
Fe	453	Ti	24
Ga	<0.1	Tl	<0.1
Ge	<0.1	U	<0.05
Hf	<0.1	V	<0.1
In	<0.1	W	<0.1
La	0.2	Y	<0.1
Li	0.2	Yb	<0.1
Mn	33.7	Zn	45.7
		Zr	0.8

**Table 5:** Amino acid composition of *L. vulgaris* Mill. aerial parts determined by GLC after acid hydrolysis (tryptophan not determined).

Amino Acids	Content (mg/100 g)	Amino Acids	Content (mg/100 g)
Alanine	862	Aspartate	1516
Glycine	283	Cystine	34
Leucine	315	Hydroxyproline	2
Isoleucine	336	Phenylalanine	316
Valine	252	Tyrosine	335
Glutamate	2820	Histidine	256
Threonine	216	Ornithine	2
Proline	480	Arginine	415
Methionine	65	Lysine	328
Serine	362		

**Table 6:** Retention times and contents of flavonoids in the 70% ethanolic extract of *L. vulgaris* Mill. determined by RP-HPLC.

Compound	RT (min)	Content (%)
Rutin (**1**)	3.84	0.37
Linarin (**2**)	4.63	3.91
Quercetin (**3**)	6.20	0.47
Apigenin (**4**)	7.41	0.19

**Table 7:** Tentatively identified constituents of the essential oil of *L. vulgaris* Mill. aerial parts obtained by hydrodistillation (GC-MS, HP-5MS column).

RT (min)	Component	RI	Content (%)
2.808	3-Methylbutan-1-ol (isopentanol)	726	0.77
4.934	(*Z*)-Hex-3-en-1-ol	856	1.17
5.246	Hexan-1-ol	868	0.8
7.788	Benzaldehyde (**5**)	958	6.75
8.452	Oct-1-en-3-ol	979	0.55
8.985	Octan-3-ol	995	0.39
10.567	Phenylacetaldehyde	1042	0.76
19.889	4-Vinylguaiacol	1313	1.08
19.977	Theaspirane B	1308	0.53
21.750	Decanoic acid	1373	0.67
21.957	α-Ylangene	1372	0.50
22.224	(*E*)-β-Damascenone	1367	0.59
22.872	Methyl eugenol	1401	0.50
23.337	Isocaryophyllene	1411	2.12
23.683	*cis*-Thujopsene	1433	1.94
24.362	Geranylacetone	1453	1.11
25.238	Germacrene D	1480	0.58
25.377	(*E*)-β-Ionone	1486	0.88
35.282	Hexahydrofarnesyl acetone (**6**)	1846	11.71
37.289	Methyl palmitate	1928	0.77
38.227	Palmitic acid (**7**)	1960	6.82
41.629	Phytol (**8**)	2112	11.53

**Table 8:** Ferric-reducing antioxidant capacity (absorbance at 700 nm) of the 70% ethanolic extract of *L. vulgaris* Mill. (LVu70) and ascorbic acid at different concentrations (mean ± SD, *n* = 3).

Sample	0.25 mg/mL	0.50 mg/mL	0.75 mg/mL	1.00 mg/mL
LVu70	0.7066 ± 0.012	0.7914 ± 0.015	0.8378 ± 0.01	1.2124 ± 0.02
Ascorbic acid	1.925 ± 0.01	2.284 ± 0.02	2.257 ± 0.01	2.316 ± 0.01

**Table 9:** DPPH radical-scavenging activity (%) of the 70% ethanolic extract of *L. vulgaris* Mill. (LVu70) and BHA at different concentrations (mean ± SD, *n* = 3).

Sample	0.25 mg/mL	0.50 mg/mL	0.75 mg/mL	1.00 mg/mL
LVu70	27.05 ± 0.82	62.50 ± 0.74	70.68 ± 0.36	76.65 ± 0.24
BHA	80.70 ± 0.02	80.30 ± 0.15	80.50 ± 0.17	80.70 ± 0.18

## Data Availability

The original contributions presented in this study are included in the article. Further inquiries can be directed to the corresponding author.

## Abbreviations

The following abbreviations are used in this manuscript:

ANOVA	Analysis of variance
BHA	Butylated hydroxyanisole
DPPH	2,2-Diphenyl-1-picrylhydrazyl
EAEU	Eurasian Economic Union
GACP	Good Agricultural and Collection Practices
GC–MS	Gas chromatography–mass spectrometry
GLC	Gas–liquid chromatography
HPLC	High-performance liquid chromatography
ICP-OES	Inductively coupled plasma optical emission spectrometry
LOD	Loss on drying
NIOCH SB RAS	N.N. Vorozhtsov Novosibirsk Institute of Organic Chemistry, Siberian Branch of the Russian Academy of Sciences
RI	Retention index
RP-HPLC	Reversed-phase high-performance liquid chromatography
SD	Standard deviation
SP RK	State Pharmacopeia of the Republic of Kazakhstan

## References

[1] Farnsworth N.R., Soejarto D.D. (1991). Global Importance of Medicinal Plants. Conservation of Medicinal Plants.

[2] Sarsenbayev K.N. (2018). Medicinally Important Plants of Kazakhstan. Vegetation of Central Asia and Environs.

[3] Ministry of Health of the Republic of Kazakhstan (2009). The State Pharmacopoeia of the Republic of Kazakhstan.

[4] Ministry of Health of the Republic of Kazakhstan (2014). The State Pharmacopoeia of the Republic of Kazakhstan.

[5] Cheriet T., Mancini I., Seghiri R., Benayache F., Benayache S. (2015). Chemical Constituents and Biological Activities of the Genus *Linaria* (Scrophulariaceae). Nat. Prod. Res..

[6] Saner M.A., Clements D.R., Hall M.R., Doohan D.J., Crompton C.W. (1995). The Biology of Canadian Weeds. 105. *Linaria vulgaris* Mill. Can. J. Plant Sci..

[7] Vasilyeva A.N., Gamayunova A.P., Dmitrieva A.A. (1965). Flora of Kazakhstan.

[8] Tekaya-Karoui A., Hammami S., Mrad N.N., Chraief I., Ben Jannet H., Mighri Z. (2010). Chemical Composition of Floral Essential Oil from *Linaria heterophylla* Desf. Growing in Tunisia. J. Essent. Oil Bear. Plants.

[9] Gruenwald J., Brendler T., Jaenicke C. (1998). PDR for Herbal Medicines.

[10] Hua H., Cheng M., Li X., Pei Y. (2002). A New Pyrroloquinazoline Alkaloid from *Linaria vulgaris*. Chem. Pharm. Bull..

[11] Ilieva E.I., Handjieva N.V., Popov S.S. (1992). Iridoid Glucosides from *Linaria vulgaris*. Phytochemistry.

[12] Ilieva E.I., Handjieva N., Spassov S., Popov S.S. (1993). 5-O-Allosylantirrinoside from *Linaria* Species. Phytochemistry.

[13] Sticher O. (1971). Isolation of Antirrinoside from *Linaria vulgaris*. Phytochemistry.

[14] Vrchovská V., Spilková J., Valentão P., Sousa C., Andrade P.B., Seabra R.M. (2008). Assessing the Antioxidative Properties and Chemical Composition of *Linaria vulgaris* Infusion. Nat. Prod. Res..

[15] Kuang W., Zhang X., Lan Z. (2018). Flavonoids Extracted from *Linaria vulgaris* Protect against Hyperlipidemia and Hepatic Steatosis Induced by Western-Type Diet in Mice. Arch. Pharm. Res..

[16] Itzhanova K.I., Nurkadirov D.K., Ishmuratova M.Y., Kosherbek A.B., Baidullayeva S.A., Poleszak E. (2024). Histochemical Analysis of Raw Materials of *Linaria vulgaris* Mill. Growing in Central Kazakhstan. Res. J. Pharm. Technol..

[17] Itzhanova K.I., Nurkadirov D.K., Ishmuratova M.Y., Abdrakhmanova G.M., Tulebayev Y.A., Kosherbek A.B. (2025). Anatomical Study of the Plant *Linaria vulgaris* Mill. Growing in Central Kazakhstan. Pharm. Kazakhstan.

[18] Nurkadirov D.K., Itzhanova K.I., Ishmuratova M.Y., Shaimerdenova Z.R. Macroscopic Characteristics of the Medicinal Plant Raw Material of *Linaria vulgaris* Mill. Proceedings of the XIII International Scientific Conference of Young Scientists “Modern Trends in the Development of Health-Preserving Technologies”.

[19] World Health Organization (2007). WHO Guidelines for Assessing Quality of Herbal Medicines with Reference to Contaminants and Residues.

[20] Gravel I.V., Shoikhet Y.N., Yakovlev G.P., Samylina I.A. (2012). Farmakognoziya: Ekotoksikanty v Lekarstvennom Rastitelnom Syre i Fitopreparatakh.

[21] Maiga A., Diallo D., Bye R. (2005). Determination of Some Toxic and Essential Metal Ions in Medicinal and Edible Plants from Mali. J. Agric. Food Chem..

[22] Luo L., Dong L., Huang Q. (2021). Detection and Risk Assessments of Multi-Pesticides in 1771 Cultivated Herbal Medicines by LC/MS-MS and GC/MS-MS. Chemosphere.

[23] European Directorate for the Quality of Medicines & HealthCare (2022). European Pharmacopoeia.

[24] Lay-Pruitt K.S., Wang W., Prom-U-Thai C., Pandey A., Zheng L., Rouached H. (2022). A Tale of Two Players: The Role of Phosphate in Iron and Zinc Homeostatic Interactions. Planta.

[25] Li M., Watanabe S., Gao F., Dubos C. (2023). Iron Nutrition in Plants: Towards a New Paradigm?. Plants.

[26] Bhattacharya S. (2026). Recent Advances in Medicinal Plant Research for Mitigating Aluminium Toxicity. J. Environ. Pathol. Toxicol. Oncol..

[27] Stanton C., Sanders D., Krämer U., Podar D. (2022). Zinc in Plants: Integrating Homeostasis and Biofortification. Mol. Plant.

[28] Pessoa C.C., Luís I.C., Daccak D.F., Legoinha P.A., Ramalho J.C., Lidon F.C., Silva M.M. (2026). Mn’s Key Roles in Plant Ecophysiology—A Comprehensive Review for Unstressed and Stress Conditions. Agronomy.

[29] Antonopoulou I., Varriale S., Topakas E., Rova U., Christakopoulos P., Faraco V. (2016). Enzymatic Synthesis of Bioactive Compounds with High Potential for Cosmeceutical Application. Appl. Microbiol. Biotechnol..

[30] Saewan N., Vichit W., Prinyarux T. (2018). Anti-Aging Efficacy of Thai Red Rice Callus Cosmetic Product. J. Appl. Sci..

[31] Qi W., Chen Y., Sun S., Xu X., Zhan J., Yan Z., Shang P., Pan X., Liu H. (2021). Inhibiting TLR4 Signaling by Linarin for Preventing Inflammatory Response in Osteoarthritis. Aging.

[32] Tian D., Yang Y., Yu M., Han Z.Z., Wei M., Zhang H.W., Jia H.M., Zou Z.M. (2020). Anti-Inflammatory Chemical Constituents of Flos Chrysanthemi Indici Determined by UPLC-MS/MS Integrated with Network Pharmacology. Food Funct..

[33] Yu Q., Li X., Cao X. (2017). Linarin Could Protect Myocardial Tissue from the Injury of Ischemia-Reperfusion through Activating Nrf-2. Biomed. Pharmacother..

[34] Mottaghipisheh J., Taghrir H., Boveiri Dehsheikh A., Zomorodian K., Irajie C., Mahmoodi Sourestani M., Iraji A. (2021). Linarin, a Glycosylated Flavonoid, with Potential Therapeutic Attributes: A Comprehensive Review. Pharmaceuticals.

[35] Agati G., Tattini M. (2010). Multiple Functional Roles of Flavonoids in Photoprotection. New Phytol..

[36] Szabados L., Savouré A. (2010). Proline: A Multifunctional Amino Acid. Trends Plant Sci..

[37] Tkachev A.V. (2008). Issledovanie Letuchikh Veshchestv Rastenij [Study of Volatile Compounds of Plants].

[38] Nafis A., Saad F.E., Khalloufi F.E., Kasrati A., Abbad A., Mezrioui N., Oudra B., Vasconcelos V., Hassani L. (2021). New Insight into Antimicrobial Activities of *Linaria ventricosa* Essential Oil and Its Synergetic Effect with Conventional Antibiotics. Arch. Microbiol..

[39] Nazlić M., Kremer D., Fredotović Ž., Trumbić Ž., Dunkić V., Puizina J. (2023). Free Volatile Compounds as Chemophenetic Markers—Comparison with ITS2 and ITS1-5.8S-ITS2 Sequence Data for 18 Species of the Genus *Veronica*. Horticulturae.

[40] Asgharian P., Heshmati Afshar F., Asnaashari S., Bamdad Moghaddam S., Delazar A. (2016). The Seasonal Variations of the Chemical Composition of Essential Oil Obtained from *Scrophularia frigida*. Jundishapur J. Nat. Pharm. Prod..

[41] Knudsen J.T., Eriksson R., Gershenzon J., Ståhl B. (2006). Diversity and Distribution of Floral Scent. Bot. Rev..

[42] Islam M.T., Ali E.S., Uddin S.J., Shaw S., Islam M.A., Ahmed M.I., Chandra Shill M., Karmakar U.K., Yarla N.S., Khan I.N. (2018). Phytol: A Review of Biomedical Activities. Food Chem. Toxicol..

[43] Wojcik M., Burzynska-Pedziwiatr I., Wozniak L.A. (2010). A Review of Natural and Synthetic Antioxidants Important for Health and Longevity. Curr. Med. Chem..

[44] Rice-Evans C., Miller N.J., Paganga G. (1996). Structure-Antioxidant Activity Relationships of Flavonoids and Phenolic Acids. Free Radic. Biol. Med..

[45] Mouffouk C., Mouffouk S., Oulmi K., Mouffouk S., Haba H. (2020). *In vitro* photoprotective, hemostatic, anti-inflammatory and antioxidant activities of the species *Linaria scariosa* Desf. S. Afr. J. Bot..

[46] Gul M., Ozturk Cali I., Cansaran A., Idil O., Kulu I., Celikoglu U. (2017). Evaluation of phytochemical content, antioxidant, antimicrobial activity and DNA cleavage effect of endemic *Linaria corifolia* Desf. (Plantaginaceae). Cogent Chem..

[47] Cheriet T., Hanfer M., Boudjelal A., Baali N., Mancini I., Seghiri R., Ameddah S., Menad A., Benayache F., Benayache S. (2017). Glycosyl flavonoid profile, *in vivo* antidiabetic and in vitro antioxidant properties of *Linaria reflexa* Desf. Nat. Prod. Res..

[48] Martínez A., Estévez J.C., Silva-Pando F.J. (2013). Antioxidant activity, total phenolic content and skin care properties of 35 selected plants from Galicia (NW Spain). Front. Life Sci..

[49] Gaamoune S. (2018). Antioxidant and antimicrobial activities of aqueous extract of *Linaria simplex* DC. Phytochem BioSub J..

[50] RSE “Kazhydromet” (2026). Climate of Karaganda: Climatic Norms for 1991–2020 (Karaganda Meteorological Station).

[51] Pogoda i Klimat [Weather and Climate]. Karaganda (WMO Station 35394): Monthly Mean Air Temperatures in 2024. http://www.pogodaiklimat.ru/history/35394.htm.

[52] Elzinga C.L., Salzer D.W., Willoughby J.W. (2019). Measuring & Monitoring Plant Populations.

[53] Ministry of Health of the Republic of Kazakhstan (2008). State Pharmacopoeia of the Republic of Kazakhstan.

[54] Losseva I., Tulebayev Y., Ishmuratova M., Kukula-Koch W., Akhmetova S., Karilkhan I., Orazbayeva P., Medeshova A., Abdrakhmanova G., Kaiyrbekova K. (2025). Evaluation of Anti-Inflammatory Activity and Chemical Composition of *Filipendula vulgaris* Moench and *Filipendula ulmaria* (L.) Maxim Extracts Obtained by Ultrasonic Method. Farmacia.

[55] Adams R.F. (1974). Determination of Amino Acid Profiles in Biological Samples by Gas Chromatography. J. Chromatogr. A.

[56] Suleimen E.M., Mamytbekova G.K., Nurkadyrov D.K., Doudkin R.V., Gorovoy P.G. (2026). Composition and Biological Activity of Essential Oil from *Peucedanum coreanum*. Chem. Nat. Compd..

[57] Ademosun A.O., Oboh G., Bello F., Ayeni P.O. (2016). Antioxidative Properties and Effect of Quercetin and Its Glycosylated Form (Rutin) on Acetylcholinesterase and Butyrylcholinesterase Activities. J. Evid. Based. Complement. Altern. Med..

[58] Tuleuov B., Zeinuldina A., Bazarkhankyzy A., Kozhanova A., Temirgaziyev B., Dyussekeyeva S., Abulyaissova L., Askarova N., Temirbekova A., Tekebayeva Z. (2026). Β-Cyclodextrin Inclusion Complexes of 20-Hydroxyecdysone Derivatives: Synthesis, NMR Characterization, and In Vitro/In Vivo Evaluation of Antioxidant, Hepatoprotective, and Antimicrobial Activities. Pharmaceuticals.

[59] Brand-Williams W., Cuvelier M.E., Berset C. (1995). Use of a Free Radical Method to Evaluate Antioxidant Activity. LWT-Food Sci. Technol..

